# Effectiveness of Nateglinide on *In Vitro* Insulin Secretion from Rat Pancreatic Islets Desensitized to Sulfonylureas

**DOI:** 10.1155/EDR.2001.73

**Published:** 2001

**Authors:** Shiling Hu, Shuya Wang, Beth E. Dunning

**Affiliations:** 1 Metabolic and Cardiovascular Diseases Novartis Institute for Biomedical Research 556 Morris Avenue Summit NJ 07901 USA

## Abstract

Chronic exposure of pancreatic islets to sulfonylureas (SUs) is known to impair the ability of islets to respond to subsequent acute stimulation by SUs or glucose. Nateglinide (NAT) is a novel insulinotropic agent with a primarily site of action at *β*-cell K_ATP_ channels, which is common to the structurally diverse drugs like repaglinide (REP) and the SUs. Earlier studies on the kinetics, glucosedependence and sensitivity to metabolic inhibitors of the interaction between NAT and K_ATP_ channels suggested a distinct signaling pathways with NAT compared to REP, glyburide (GLY) or glimepiride (GLI). To obtain further evidence for this concept, the present study compared the insulin secretion *in vitro* from rat islets stimulated acutely by NAT, GLY, GLI or REP at equipotent concentrations during 1-hr static incubation following overnight treatment with GLY or tolbutamide (TOL). The islets fully retained the responsiveness to NAT stimulation after prolonged pretreatment with both SUs, while their acute response to REP, GLY, and GLI was markedly attenuated, confirming the desensitization of islets. The insulinotropic efficacy of NAT in islets desensitized to SUs may result from a distinct receptor/effector mechanism, which contributes to the unique pharmacological profile of NAT.


*Int. Jnl. Experimental Diab. Res.*, Vol. 2, pp. 73–79
Reprints available directly from the publisher
Photocopying permitted by license only

© 2001 OPA (Overseas Publishers Association) N.V.
Published by license under
the Harwood Academic Publishers imprint,
part of Gordon and Breach Publishing,
a member of the Taylor & Francis Group.
Printed in the United States.

### Effectiveness of Nateglinide on *In Vitro* Insulin Secretion from Rat Pancreatic Islets Desensitized to Sulfonylureas

SHILING HU*, SHUYA WANG and BETH E. DUNNING

*Metabolic and Cardiovascular Diseases, Novartis Institute for Biomedical Research, Summit, USA*

*(Received 6 December 2000; In final form 5 February 2001)*

**Chronic exposure of pancreatic islets to sulfonylureas (SUs) is known to impair the ability of islets to respond to subsequent acute stimulation by SUs or glucose. Nateglinide (NAT) is a novel insulinotropic agent with a primarily site of action at $\beta$-cell $K_{ATP}$ channels, which is common to the structurally diverse drugs like repaglinide (REP) and the SUs. Earlier studies on the kinetics, glucose-dependence and sensitivity to metabolic inhibitors of the interaction between NAT and $K_{ATP}$ channels suggested a distinct signaling pathways with NAT compared to REP, glyburide (GLY) or glimepiride (GLI). To obtain further evidence for this concept, the present study compared the insulin secretion *in vitro* from rat islets stimulated acutely by NAT, GLY, GLI or REP at equipotent concentrations during 1-hr static incubation following overnight treatment with GLY or tolbutamide (TOL). The islets fully retained the responsiveness to NAT stimulation after prolonged pretreatment with both SUs, while their acute response to REP, GLY, and GLI was markedly attenuated, confirming the desensitization of islets. The insulinotropic efficacy of NAT in islets desensitized to SUs may result from a distinct receptor/effector mechanism, which contributes to the unique pharmacological profile of NAT.**

*Keywords:* Rat pancreatic islet; Static incubation; *In vitro* insulin secretion; Nateglinide; Islet desensitization

### INTRODUCTION

Sulfonylureas (SUs) are widely used in the treatment of non-insulin-dependent (Type 2) diabetes. They stimulate insulin secretion by closing $\beta$-cell plasma membrane $K_{ATP}$ channels,[1] which are formed by the molecular interaction between a high-affinity SU receptor, SUR1, and an inwardly rectifying $K^+$ channel subunit, Kir6.2.[2] The closure of $K_{ATP}$ channels leads to the opening of voltage-dependent $Ca^{2+}$ channels, $Ca^{2+}$ influx and stimulation of $Ca^{2+}$-dependent exocytosis.[3] While SUs exert hypoglycemic action *via* a direct stimulation of insulin release during short term administration in type 2 diabetic patients, their activity declines during long term applica-tion, which has been suggested to be directly attributable to a desensitization of $\beta$-cells to SUs.[4–7] *In vitro* chronic exposure of pancreatic islets to SUs is also known to cause impairment of secretory response to subsequent stimulation by glucose or SUs.[8–11] The mechanisms underlying

*Address for correspondence: Metabolic and Cardiovascular Diseases, Novartis Institute for Biomedical Research, 556 Morris Avenue, Summit, NJ 07901, USA. Tel.: 908-277-5703, Fax: 908-277-4756, e-mail: shiling.hu@pharma.novartis.com

$\beta$-cell refractoriness after prolonged exposure to SUs remain at present unclear.

Nateglinide (NAT), a novel D-phenylalanine derivative, shares the mechanism of action with SUs to act on $K_{ATP}$ channels in pancreatic $\beta$-cells. This drug, compared to other marketed $K_{ATP}$ channel-blocking hypoglycemic agents, has demonstrated unique characteristics including a rapid onset, short duration of action, sensitivity to ambient glucose, and resistance to metabolic inhibition, suggesting some aspects of the signaling pathway(s) mediating NAT's action are novel and distinct from those mediating the effects of SUs and REP. This study was designed to evaluate the ability of NAT and the com-parators, glyburide (GLY), glimepiride (GLI), and repaglinide (REP) to stimulate insulin secretion from isolated rat islets undergoing prolonged pre-exposure to SUs, GLY or tolbutamide (TOL). Using static incubation method, normal rat islets were incubated overnight (~18hours) in the absence and presence of therapeutically relevant concentrations of GLY or TOL before their acute responsiveness to NAT, GLY, REP, and GLI was determined. Our results showed that lasting treatment of GLY or TOL markedly attenuated the islets' acute response to GLY, GLI and REP, while NAT retained ability to stimulate insulin secretion from SU-desensitized islets. These findings are suggestive of common signaling pathways for the action of SUs and REP. On the other hand, the effectiveness of NAT in SU-desensitized islets could imply the involvement of an action site and/or a receptor/ effector pathway different from those of SUs and REP. The unique "desensitization-resistant" properties of NAT may explain some of the pharmacological differences between those of NAT and those of SUs and REP.[12,13]

### MATERIALS AND METHODS

### Islet Isolation

Pancreas were dissected from normal fed male Sprague Dawley rats (250–275g), which were euthanized with Na pentobarbital i.p. at 120mg/kg. Islets of Langerhans were isolated by librase digestion (0.5mg/ml, Boehringer Mannheim, Germany) followed by a Ficoll gradient centrifugation.[14]

### Static Incubation of Islets Desensitized to SUs

Freshly isolated islets were handpicked under a stereomicroscope by gentle suction through a large firepolished pipette (~400 μm diameter) into 60×15 mm Petri dishes (Corning) containing 25ml of DMEM (Dulbecco's Modified Eagle Medium, Gibco BRL) supplemented with 10% fetal calf serum, 5mM glucose (G5-DMEM) and 1% BSA (BSA was present in all incubation media throughout the experiments). Islets were preincubated in a humidified atmosphere of 95% $O_2$ and 5% $CO_2$ at 37°C for 1 hour.

The islets, after 1 hour preincubation, were selected in batches of two and placed into borosilicate glass tubes (12×75mm) containing 500 μl G8-DMEM in the presence or absence of 100nM or 10 μM GLY or 100 μM TOL, and incubated over night (~18hours) in a humidified atmosphere of 95% $O_2$/5% $CO_2$ at 37°C. Acute experiments were performed the following morning to determine the ability of islets to respond to NAT and other insulinotropic agents. 500 μl 2x treatment of the concentrations of test hypoglycemic drugs was added to the tubes (to form 1 ml final volume) containing either GLY/TOL-treated or GLY/ TOL-untreated islets. The concentrations of test hypoglycemic drugs were so chosen that they were comparably effective in control, *i.e.*, 5 μM NAT, 100nM GLY, 100nM GLI and 50nM REP. All test drugs were first dissolved in DMSO to form a stock solution of 10 or 30mM and stored at 4°C. The stock solutions were further diluted with incubation media to the desired concentrations before each experiment. Eight independent samples were run for each

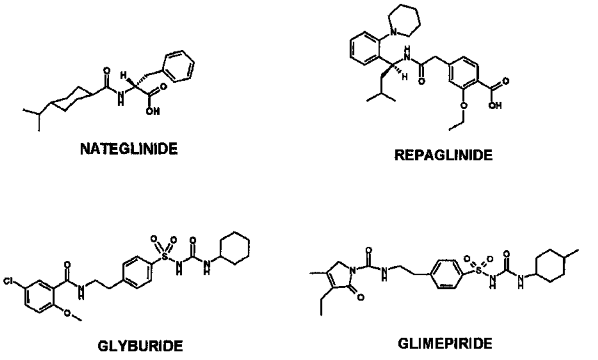

FIGURE 1 Chemical structure of test antidiabetic drugs.

experimental condition. Tubes were incubated at 37°C with intermittent hand shaking for 1hour. At the end of incubation, islet media (500 μl/ tube) were transferred to 96 deep-well plates and stored at −20°C for subsequent insulin analysis.

### Insulin Scintillation Proximity Assay (SPA)

The incubation media were diluted by factors ranging from lx to 20x depending on the concen-trations of glucose/drugs. The diluted super-natant was assayed for insulin content with SPA.[15] The assay employed commercially available products including a guinea pig antirat insulin specific antibody (Linco Research Inc) and scin-tillation proximity Type I reagent coupled to protein A (Amersham Life Science), and was performed as a single step assay. All samples were assayed in duplicate. The preparation of 96 well sample plates was made by sequentially adding standard/test samples, anti-insulin serum, [$^{125}$I]-insulin tracer, and SPA reagent, and the final volume equaled 175 μl/ well. The plates were incubated and vortexed on a titer plate shaker for approximately 16 hours at room temperature. The plates were then placed into a Wallac Microbeta 1450 Liquid Scintillation Counter to be read under a normalization protocol. The output was in counts per minute (CPM).

### Data Analysis

The sample insulin concentration was calculat-ed by utilizing a template set up in Excel spreadsheet that possesses statistical analysis function. The calculated concentration was adjusted eventually to reflect the degree of dilution. The intra- and inter-assay coefficients of variation were generally between 5% and 8%. To allow comparison of the hypoglycemic drug-induced insulin secretion from overnight SU-treated and untreated islets, data were expressed as percent of the appropriate con-trols. That is to say, insulin secretion stimulat-ed by secreta-gogues in SU-treated and untreated groups was normalized, respectively, with the amount of insulin without secreta-gogues in SU-treated and untreated groups. This transformation cor-rected for the increase of control values elicited by overnight treat-ment of SUs. All values were expressed as the mean ± SEM. Statistical signifi-cance was determined with t-test (single-tailed). $P < 0.05$ was considered significantly different.

### RESULTS

### Drug-induced Insulin Secretion from Islets with Overnight Treatment of 100nM GLY

It was assumed that prolonged treatment with SUs would impair islet responsiveness to physiological and pharmacological stimuli. The effectiveness of four hypoglycemic agents, NAT, GLY, GLI, and REP, to stimulate insulin release from islets cultured overnight (18 hours) in the presence of 100nM GLY was evaluated and was compared to that from overnight cultured islets without GLY to determine the magnitude of desensitization induced by GLY. In the acute experiments following overnight incubation, the concentrations of test drugs were so chosen that they were comparably effective in stimulating insulin release at glucose concentration of 8mM, *i.e.*, NAT at 5 μM, GLY at 100nM, GLI at 100nM, and REP at 50nM. These hypoglycemic agents stimulated insulin secretion during 1 hour static incubation from islets cultured overnight without 100nM GLY by a respective 270 ± 44%, 292 ± 42%, 301 ± 26% and 430 ± 35%. In parallel experiments on islets cultured overnight with 100nM GLY, the stimulation factors were, respectively, 354 ± 19%, 302 ± 13%, 340 ± 12%, and 352 ± 7%. These data are illustrated in Figure 2, in which the stimulation factors of all test agents were not statistically different between islet groups without and with pretreatment of 100nM GLY. Thus, prolonged treatment with glyburide at 100nM did not appear to render the islets insensitive to subsequent application of insulin secretagogues.

### Drug-induced Insulin Secretion from Islets with Overnight Treatment of 10 μM GLY

When the concentration of GLY for overnight treatment was raised by 100-fold to 10 μM, the pattern of the acute response of islets to insulin secretagogues has changed and the results are shown in Figure 3. The ability of NAT to stimulate insulin secretion did not significantly differ between the GLY untreated control (126 ± 20%) and the GLY pretreated groups (155 ± 14%). Conversely, the acute stimulation factors by GLY, REP and GLI in the groups without or

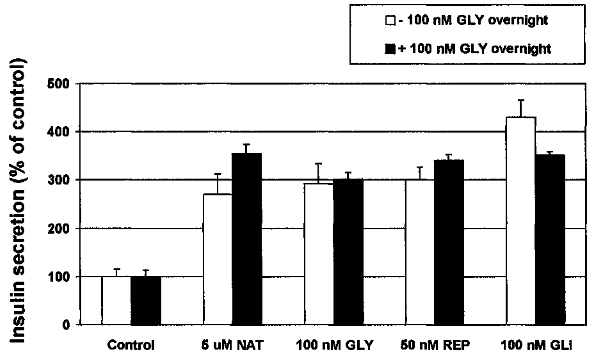

FIGURE 2 Insulin release during 1 hour static incubation in response to acute challenges by antidiabetic agents from rat islets following overnight treatment without (empty bars) and with (filled bars) 100nM GLY at G8. Data are expressed as % of appropriate controls ( ± GLY, n = 16) *p < 0.05 when compare between data with and without pretreatment.

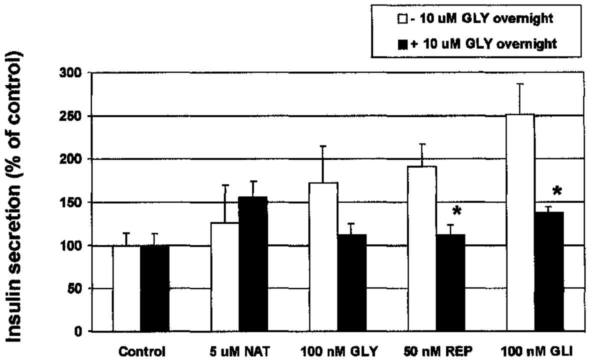

FIGURE 3 Insulin release during 1 hour static incubation in response to acute challenge by antidiabetic agents from rat islets following overnight treatment without (empty bars) and with (filled bars) 10 μM GLY at G8. Data were pooled from 16 independent samples/condition and expressed as % of appropriate controls ( ± GLY). *p < 0.05 when compare between data with and without pretreatment. Basal insulin secretion without and with GLY pretreatment was, respectively, 265 ± 25 and 413 ± 62 μU/islet/1h.

with 10 μM GLY pretreatment were, respectively, 173 ± 21% or 112 ± 15% (NS) with GLY, 191 ± 18% or 112 ± 11% (p < 0.05) with REP, 251 ± 33% or 138 ± 17% (p < 0.05) with GLI. Thus, the ability of GLY, REP and GLI to stimulate insulin secretion was attenuated following lasting treatment of islets with 10 μM GLY compared to that without pretreatment. The data demonstrated that 10 μM GLY induced islet desensitization leading to hypo-responsiveness of islets to the subsequent application of GLY, REP and GLI. NAT was the only agent that was able to maintain its insulinotropic efficacy intact in desensitized islets.

### Drug-induced Insulin Secretion from Islets with Overnight Treatment of 100 mM TOL

To further confirm SU induction of islet desensitization and the effectiveness of NAT, tolbutamide (TOL), a classic first generation SU, was also used as a desensitizing agent for the assessment of the ability of four hypoglycemic drugs on secretory response of islets. The stimulation factors of the test drugs during 1 hour static incubation following overnight

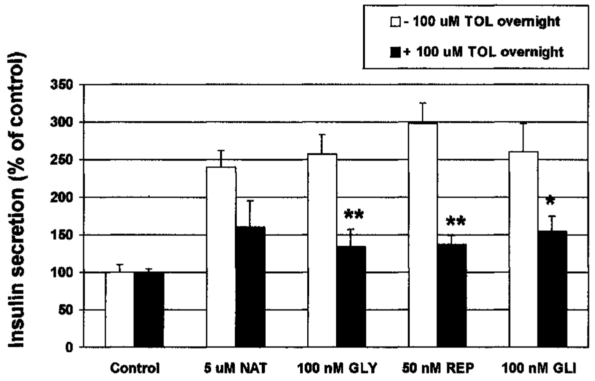

FIGURE 4 Insulin release during 1 hour static incubation in response to acute challenge by antidiabetic agents from rat islets following overnight treatment without (empty bars) and with (filled bars) 100 μM TOL at G8. Data were pooled from 16 independent samples/condition and expressed as % of appropriate controls (± TOL). $^*p < 0.05$ and $^{**}p < 0.005$ when compare between data with and without pretreatment. Basal insulin secretion during static incubation without and with TOL pretreatment was, respectively, 210 ± 22 and 314 ± 16 μU/islet/lh.

treatment of islets with or without 100 μM TOL are illustrated in Figure 4. NAT was able to stimulate insulin by 240 ± 23% in control and 160 ± 35 after TOL pretreatment. This change was not statistically significant. On the other hand, the stimulation factors in control or TOL-pretreated islet groups were, respectively, 257±27% or 134±23% with GLY, 298±27% or 137±13% with REP, and 260±38% or 155±20% with GLI. The results reinforced the argument that islets desensitized to SUs retain responsiveness to NAT but not GLY, REP and GLI. The changes of insulin secretion in both GLY and TOL pretreated groups are summarized in the Table I.

### DISCUSSION

The present study demonstrated that prolonged exposure of pancreatic islets to SUs, GLY or -TOL, impaired their ability to secrete insulin in response to subsequent secretagogue stimulation – a phenomenon known as islet desensitization. Similar results have been previously reported for both drugs.[8–10,16] As $K_{ATP}$ channel blockade plays key role in the mechanism of action of SUs and all test drugs in this study, one possible mechanism for SU desensitization is that chronic exposure to SUs would result in lasting binding of SU to SUR1 and closing of $K_{ATP}$ channels, and in turn, a lasting membrane depolarization. The constant depolarization would lead the β-cells to a refractory state, in which they respond less effectively to a further stimulation of closing $K_{ATP}$ channels by test secretagogues. Evidence for this mechanism of SU desensitization associated with chronic exposure to GLY has been previously reported.[11] If this were indeed the case, SU desensitization is likely to be a temporary and reversible condition of refractoriness of the β-cells to secretagogue stimulation, since $K_{ATP}$ channel activity would eventually recover upon withdrawal of SUs.

The key finding in the study was that NAT was the only drug of four test agents which maintained its insulinotropic efficacy in islets desensitized to SUs. Given that islets were still able to vigorously respond to NAT after 18h pretreatment of SUs, the exhaustion of a finite insulin store and/or islet secretory machinery did not appear to be the underlying defect in

TABLE I Insulin secretion during 1h static incubation following overnight treatment of SUs (% of control)

| | 5μMNAT | 100nM GLY | 50nM REP | 100nM GLI |
|---|---|---|---|---|
| −10 μM GLY overnight | 126 ± 20 | 173 ± 21 | 191 ± 18 | 251 ± 33 |
| +10 μM GLY overnight | 155 ± 14 | 112 ± 15 | 112 ± 11* | 138 ± 17* |
| −100 μM TOL overnight | 240 ± 23 | 257 ± 27 | 298 ± 27 | 260 ± 38 |
| +100 μM TOL overnight | 160 ± 35 | 134 ± 23** | 137 ± 13** | 155 ± 20* |

All data in SU-pretreated and non-pretreated groups are expressed, respectively, as percent of insulin secretion in appropriate control groups without acute stimulation by secretagogues.
$^*p < 0.05$ and $^{**}p < 0.005$ on data with SU treatment compared to those without.

desensitized secretion to GLY, REP and GLI. The insulinotropic action of REP, GLY and GLI was desensitized by lasting SU exposure, suggestive of common site of action for these drugs. On the other hand, the inability of both TOL and GLY to affect NAT-induced insulin secretion suggested a distinct mode of action of NAT. Provided that the mechanism of desensitization was indeed associated to the refractoriness of SUR1/$K_{ATP}$ channels in $\beta$-cells, a possible explanation for the effectiveness of NAT *vs.* ineffectiveness of GLY, REP and GLI in stimulation of insulin following prolonged pretreatment of SUs would be the existence of distinct binding sites on SUR1 for NAT *vs.* SUs and REP. It is possible that the site on SUR1 for SUs has become hypo-responsive to GLY, GLI and REP due to its desensitization induced by chronic treatment of GLY or TOL, while the site on SUR1 for NAT remained fully available to be activated with or without pretreatment of SUs. Alternatively, the present data would be reconciled by proposing a second mechanism of the insulinotropic action of NAT that is independent of $K_{ATP}$ channels.

Being cautious not to over-interpret the *in vitro* findings in the present study, we only speculate that some aspects of signaling pathways mediating NAT's effect are markedly dissimilar from those involving in the effects of SUs and REP. Although the present study did not deal with time-dependent secretory pattern but cumulative stimulation of insulin secretion from islets during certain period of time, earlier works by others and us reveale essential differences of NAT action from those of GLY, GLI and REP with respect to (1) the kinetics of the interaction of NAT with SUR1 receptor/$K_{ATP}$ channels;[17] (2) the kinetics of *in vitro/in vivo* insulinotropic action;[18-22] (3) the glucose-dependence;[23,24] and (4) the sensitivity to metabolic inhibitors.[25] These findings collectively indicated the uniqueness of NAT action and possibly a distinct receptor/effector system(s). By virtue of these properties, NAT is able to ameliorate postprandial hyperglycemia by augmenting early insulin release, while SUs and REP may preferentially decrease postabsorptive hyperglycemia due to their slower onset and sustained duration of action.

In conclusion, the maintenance of insulinotropic efficacy of NAT in islets desensitized to SUs adds to the list of properties of this agent that distinguish it from other SUR ligands, despite the presumed common basic mechanism of action of said agents.

### References

[1] Aguilar-Bryan, L. and Bryan, J., Molecular biology of adenosine triphosphate-sensitive potassium channels, *Endocr. Rev.*, **20**, 101–135.

[2] Inagaki, N., Gonoi, T., Clement, J. P., Namba, N., Inazawa, J., Gonzalez, G., Aguilar-Bryan, L., Seino, S. and Bryan, J. (1995). Reconstitution of $I_{KATP}$: An inward rectifier subunit plus the sulfonylurea receptor, *Science*, **270**, 1166–1170.

[3] Ashcroft, F. M. and Rorsman, P. (1989). Electrophysiology of the pancreatic $\beta$-cell, *Prog. Biophys. Mol. Biol.*, **54**, 87–143.

[4] Dunbar, J. C. and Foa, P. P. (1974). An inhibitory effect of tolbutamide and glibenclamide on the pancreatic islets of normal animals, *Diabetologia*, **10**, 27–32.

[5] Filipponi, P., Marcelli, M., Nicoletti, I., Pacifici, R., Santeusanio, F. and Brunetti, P. (1983). Suppressive effect of long-term sulfonylurea treatment on A, B, and D-cells of normal rat pancreas, *Endocrinology*, **113**, 1972–1979.

[6] Karam, J. H., Sanz, E., Salomon, E. and Nolte, M. S. (1986). Selective unresponsiveness of pancreas B-cells to acute sulphonylurea stimulation during sulphonylurea therapy in NIDDM, *Diabetes*, **35**, 1314–1320.

[7] Grunberger, G. (1993). Continuous *versus* intermittent sulphonylurea therapy in non-insulin-dependent diabetes mellitus, *Drug Safety*, **9**, 249–253.

[8] Bolaffi, J. L., Heldt, A., Lewis, L. D. and Grodsky, G. M. (1986). The third phase of *in vitro* insulin secretion: evidence for glucose insensitivity, *Diabetes*, **35**, 370–373.

[9] Zawalich, W. S. (1989). Phosphoinoside hydrolysis and insulin secretion in response to glucose are impaired in isolated rat islets by prolonged exposure to the sulphonylurea tolbutamide, *Endocrinology*, **125**, 281–286.

[10] Gullo, D., Rabuazzo, A. M., Vetri, M., Gatta, C., Vinci, C., Buscema, M., Vigneri, R. and Purrello, F. (1991). Chronic exposure to glibenclamide impairs insulin secretion in isolated rat pancreatic islets, *J. Endocrinol. Invest.*, **14**, 287–291.

[11] Rabuazzo, A. M., Buscema, M., Vinci, C., Caltabiano, V., Vetri, M., Forte, F., Vigneri, R. and Purrello, F. (1992). Glyburide and tolbutamide induce desensitization of insulin release in rat pancreatic islets by different mechanisms, *Endocrinology*, **131**, 1815–1820.

[12] Kahn, S. E., Montgomery, B., Howell, W. M., Ligueros-Saylan, M., Hsu, C. H., Devineni, D., Mcleod, J. F. and Horowitz, A. D. (2000). Nateglinide increases first phase insulin secretion and enhances glucose clearance in Type 2 diabetes, *Diabetes*, **49**, A113.

[13] Hollander, P. A., Schwartz, S. L., Gatlin, M. R., Haas, S., Zheng, H., Foley J. E. and Dunning, B. E. (2000). Nateglinide, but not glyburide, selectively enhances early insulin release and more effectively controls post-meal glucose excursions with less total insulin exposure, *Diabetes*, **49**, A111.

[14] Lacy, P. E. and Kostianovsky, M. (1967). Method for the isolation of intact islets of Langerhans from the rat pancreas, *Diabetes*, **16**, 35–39.

[15] Cook, N. D. (1996). Scintillation proximity assay: a versatile high-throughput screening technology, *Drug Disc. Trends*, **1**, 287–294.

[16] Henquin, J. C. (1980). Tolbutamide stimulation and inhibition of insulin release: studies of the underlying ionic mechanisms in isolated rat islets, *Diabetologia*, **18**, 151–160.

[17] Hu, S., Wang, S., Fanelli, B., Bell, P. A., Dunning, B. E., Geisse, S., Schmitz, R. and Boettcher, B. R. (2000). Pancreatic $\beta$-cell KArT channel activity and receptor binding studies with nateglinide: a comparison with sulphonylureas and repaglinide, *J. Pharmacol. Exp. Ther.*, **293**(2), 444–452.

[18] Leclercq-Meyer, V., Ladriere, L., Fuhlerdorff, J. and Malaisse W. J. (1997). Stimulation of insulin and somatostatin release by two meglitinide analogs, *Endocrine*, 7(3), 311–317.

[19] Tsukuda, K., Sakurada, M., Niki, I., Oka, Y. and Kikuchi M. (1998). Insulin secretion from isolated rat islets induced by the novel hypoglycemic agent A-4166, a derivative of D-phenylalanine, *Horm. Metab. Res.*, **30**, 42–49.

[20] Dunning, B. E. and Paladini, S. (1999). Mimicking cephalic insulin release with the rapid onset/short duration insulinotropic agent, nateglinide, reduces prandial glucose excursions without increasing total insulin exposure in IGT monkeys, *Diabetes*, **48**(1), A282.

[21] Dunning, B. E. and Gutierrez, C. (1999). Pharmacodynamics of nateglinide and repaglinide in cynomolgus monkeys, *Diabetes*, **48**(1), A104.

[22] DeSouza, C. J., Gagen, K., Chen, W. and Dragonas, N., Superior glycemic control with early insulin release v/s total insulin release in thpe-2 diabetic rodent models, *Diabetes*, **48**(1), A270.

[23] Seto, Y., Fujita, H., Dan, K., Fujita, T. and Kato, R. (1995). Stimulating activity of A-4166 on insulin release in *in situ* hamster pancreatic perfusion, *Pharmacology*, **51**, 245–253.

[24] Hu, S. and Wang, S. (1998). Effect of antidiabetic agent, netaglinide, on KATP channel in $\beta$-cells: comparison to glyburide and repaglinide, *Diabetologia*, **41** Suppl., A139.

[25] Fujitani, S., Okazaki, K. and Yada, T. (1997). The ability of a new hypoglycemic agent, A-4166, compared to sulphonylureas, to increase cytosolic $Ca^{2+}$ in pancreatic $\beta$-cells under metabolic inhibition, *Br. J. Pharmacol.*, **120**, 1191–1198.

